# Supplementary material for: A Novel Genotype and First Record of Trypanosoma lainsoni in Argentina
Source: Pathogens. 2020 Sep 4;9(9):731. doi: 10.3390/pathogens9090731 (PMC7558950; doi:10.3390/pathogens9090731)
Supplement: Supplementary file 1 [file pathogens-09-00731-s001.zip › pathogens-902671supp.pdf]

pathogens

MDPI

Supplementary Materials

Table S1. Analysis of the sequences of the 18S rDNA genes (2659 bp) and gGAPDH (528 bp) obtained from the Le29 isolate in BLAST (Basic Local Alignment Search Tool). Including species belonging to the lizard and snake/rodent and marsupial (LSRM) clade.

	**18S rDNA**			gGAPDH		
**Species**	**Sites**	**Query Cover**	**Identities**	**Site**	**Query Cover**	**Identities**
Trypanosoma lainsoni	2118/2135	99%	99.2%	518/521	100%	99.42%
Trypanosoma freitasi	1989/2079	98%	95.67%	473/521	100%	90.79%
Trypanosoma gennarii	2003/2082	96%	96.2%	469/521	100%	90.02%
Trypanosoma cascavelli	2020/2139	98%	94.43%	479/521	100%	91.94%
Trypanosoma serpentis	1948/2009	93%	96.96%	478/521	100%	91.74%

Table S2. Values of genetic distance of the species of the genus *Trypanosoma* of concatenated alignment between the 18S rDNA and gGAPDH genes in percentage %, calculation of the p-corrected value by means of the MEGA program. The light blue squares represent genetic distances less than 1% and in green, the genetic distance between Le 29 and *T. lainsoni.*

	**1**	**2**	**3**	**4**	**5**	**6**	**7**	**8**	**9**	**10**	**11**	**12**	**13**	**14**	**15**	**16**	**17**	**18**	**19**	**20**	**21**
Species of the Genus *Trypanosoma*	Le29	*T. lainsoni*	*T. serpentis*	*T. cascavelli*	*T. sp. Gecko*	*T. varani*	*T. rotatorium*	*T. mega*	*T. vivax*	*T. brucei rhodesiense*	*T. evansi*	*T. simiae*	*T. congolense*	*T. theileri*	*T. avium*	*T. lewisi*	*T. microti*	*T. cruzi*	*T. cruzi marinkellei*	*T. rangeli*	*T. vespertilionis*
1		0.6	2.6	2.5	5.4	5.2	7.6	7.1	10.9	9.1	9.3	10	9.8	5.9	6.1	6.6	6.3	6.4	6.4	6.9	6.6
2			2.7	2.6	5.5	5.4	7.7	7.3	11.2	9.1	9.4	10.1	9.8	6.1	6.2	6.6	6.3	6.6	6.6	7.1	6.8
3				0.2	5.1	5.2	7.5	7.2	11	9.3	9.6	9.8	9.7	5.9	6.2	6.5	6.2	6.6	6.5	7.5	6.6
4					5	5.1	7.3	7.1	10.9	9.2	9.5	9.7	9.4	5.8	6.1	6.2	6	6.5	6.3	7.2	6.4
5						2.7	6.7	6.6	11.6	9.1	9.4	10.2	9.9	5.4	5.7	6.1	5.9	5.7	5.6	6.8	6.6
6							6.6	6.6	11.1	9.2	9.4	10.1	9.6	5.1	5.7	6	5.9	5.7	5.3	6.6	6.5
7								1.7	11.1	9	9.2	8.6	8.3	5.9	6	5.6	5.2	5.6	5.5	6.4	5.8
8									10.8	8.8	9	8.6	8.4	5.3	5.6	5	4.8	5.1	5	5.9	5
9										10	10.1	9.2	9.3	10.1	10.7	10.3	10.1	10.3	10.3	10.9	10.7
10											0.3	5.7	5.6	8.9	8.8	8.7	8.6	8.9	8.8	9.5	9
11												5.9	5.8	9.1	9.1	9	8.9	9.1	9.1	9.8	9.3
12													1.8	9	8.7	8.6	8.6	8.4	8.7	9.4	8.8
13														8.9	8.7	8.3	8.4	8.3	8.6	9.1	8.4
14															5.1	5	4.7	4.6	4.2	5.6	5.4
15																5.2	4.8	5.2	5.4	6.7	6.1
16																	0.7	4.7	4.5	5.6	4.9
17																		4.4	4.1	5.2	4.7
18																			0.7	4.1	3.6
19																				4,2	3,5
20																					3
21																					

Table S3. Values of genetic distance of the *T. cruzi* strains: *T. cruzi* Esmeraldo, *T. cruzi* Silvio, *T. cruzi* marinkellei B7, and *T. cruzi* MT4167.

	***T. cruzi* Strains**	**1**	**2**	**3**	**4**
1	*T. cruzi* Silvio				
2	*T. cruzi* Esmeraldo	0.9			
3	*T. cruzi marinkellei* B7	1.0	0.4		
4	*T. cruzi* MT4167	0.7	0.6	0.8	

Table S4. This table shows the external and internal primers used in the sequencing of the 18S rDNA gene.

**Primers**	**Sequence**
External Primers	
18sp3pL	5′-TGCTTGTTTCAAGGACTTAGC-3′
18sp3pR	5′-TACGCTTTTGGAGCTGGAAT-3′
Internal Primers	
18sSEQ1F	5′-CCCAGAACATTGAGGAGCAT-3′
18sSEQ1R	5′-TGGGAAAACCACGGAATCAC-3′
18sSEQ2F	5′-GCGTGAAGAAGTAAGAAAACGTG-3′
18sSEQ2R	5′-TATCCTCAGCACGTTTTCTTACTT-3′
18sSEQ3F	5′-GCTCCTTTGTTATCCCATGC-3′
18sSEQ3R	5′-CATGGGATAACAAAGGAGCAG-3′
18sSEQ4F	5′-ACTGGCTGATAGGGCAGTTG-3′
18sSEQ4R	5′-GGAGAGGGAGCCTGAGAAAT-3′

Table S5. Hosts and geographic origin of trypanosomatids and GenBank accession numbers of sequences included in this study.

**Species**	**Origin**		**GenBank Accession Number**	
	**Host**	**Geographic Location**	**gGAPDH**	**18S rDNA**
T. lainsoni	*Mesomys* sp.	Brazil	MF406153.1	MF403111.1
T. freitasi	*Didelphis albiventris*	Brazil	MF401952.1	MF401951.1
T. cascavelli	*Crotalus durissus terrificus*	Brazil	FJ236511.1	EUO95837
T. serpentis	*Pseudoboa nigra*	Brazil	FJ236512.1	EUO95839
T. sp. Gecko	*Tarentola annularis*	Senegal	AJ620259.1	AJ620548.1
T. varani	*Varanus exanthematicus*	Senegal	AB362559.1	AJ005279.1
T. avium	*Chaffinch Fringilla coelebs*	Czech Republic	AJ620263.1	AJ009140.1
T. theileri	*Bos taurus*	Germany	KU587664.1	AJ009163.1
T. cyclop	*Macaca nemestrina*	Malaysia	-	AJ131958.1
T. pestanai	*Meles meles*	France	AJ620275.1	AJ009159.1
T. vivax	*Bos Taurus*	Africa	AF053744.1	U22316.1
T. brucei rhodensiese	*Homo sapiens*	Zambia	AJ620284.1	KX007997.1
T. evansi	*Hydrochoerus hydrochaeris*	Brazil	AF053743.1	KY114577.1
T. simiae	*Glossina m. submorsitans*	Gambia	AJ620293.1	AJ009162.1
T. congolense	*Bos Taurus*	Gambia	AJ620291.1	U22318.1
T. lewisi	*Rattus rattus*	England	AJ620272.1	AJ009156.1
T. microti	*Microtis agrestis*	England	AJ620273.1	AJ009158.1
T. livingstonei	*Hipposideros caffer*	Mozambique	KF192969.1	KF192983.1

T. cruzi marinkelei	*Phyllostomus discolor*	Brazil	AJ620270.1	AJ009150.1
T. cruzi	Mammals	America	MG471429.1	AF288661.1
T. erneyi	*Tadarida* sp.	Mozambique	JN040964.1	JN040988.1
T. rangeli	Mammals	Central and South America	AF053742.1	AY491767.1
T. vespertilionis	*Pipistrellus pipistrellus*	England	AJ620283.1	AJ009166.1
T. conhorini	*Rattus rattus*	Brazil	AJ620267.1	AJ012411.1
T. dionisii	*Pipistrellus pipistrellus*	England	MH393937	AJ009151.1
T. binneyi	*Bufo regularis*	Canada	AJ620266.1	-
T. rotatorium	*Rana catesbeiana*	Canada	AJ620256.1	AJ009161.1
T. mega	*Bufo regularis*	Africa	AJ620253.1	AJ009157
H. megaselie	*Megaselia scalaris*	USA	JQ359749.1	U01014.1
H. muscarum	*Musca domestica*	USA	JQ359748.1	L18872.1
H. samuelpessoai	*Chrysomya putoria*	Guinea-Bisáu	JQ359732.1	U01016.1
C. fasiculata	*Anopheles maculipennis*	Unknown	AF053739.1	Y00055.1
C. brevicula	*Nabis brevis*	Europe	KJ443342.1	MT232051.1
L. brasiliensis	*Homo sapiens*	Brazil	-	JX030135.1
L. tarentolae	*Tarentola mauritanica*	Algeria	KX790726.1	-

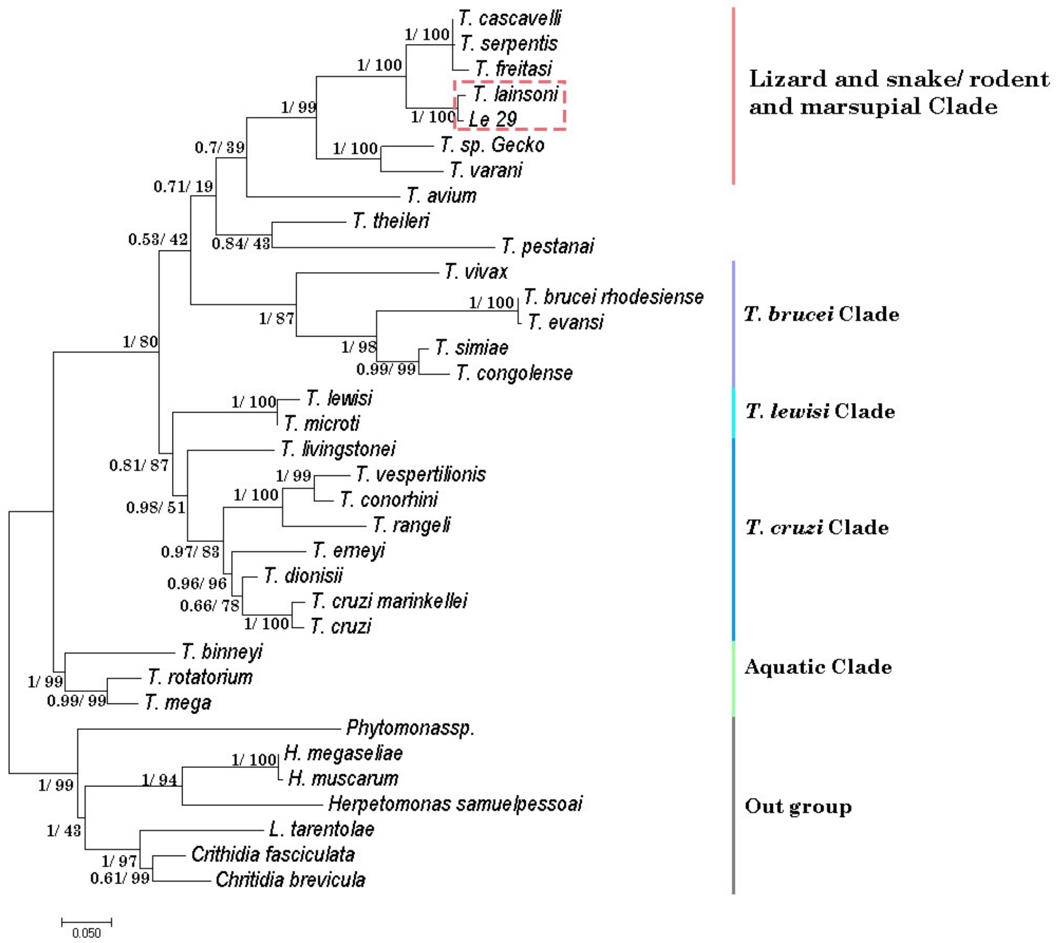

Figure S1. Phylogenetic reconstruction performed by the maximum likelihood method from 528 bp of the gGAPDH gene in 35 species of trypanosomatids. Species of the genera *Herpetomonas*, *Crithidia*, *Leishmania*, and *Phytomonas* were included as *out* groups. The two values at the nodes represent: Bayesian analysis support/ML method bootstrap value. *Trypanosoma lainsoni* and Le29 are found within the clade LSRM.

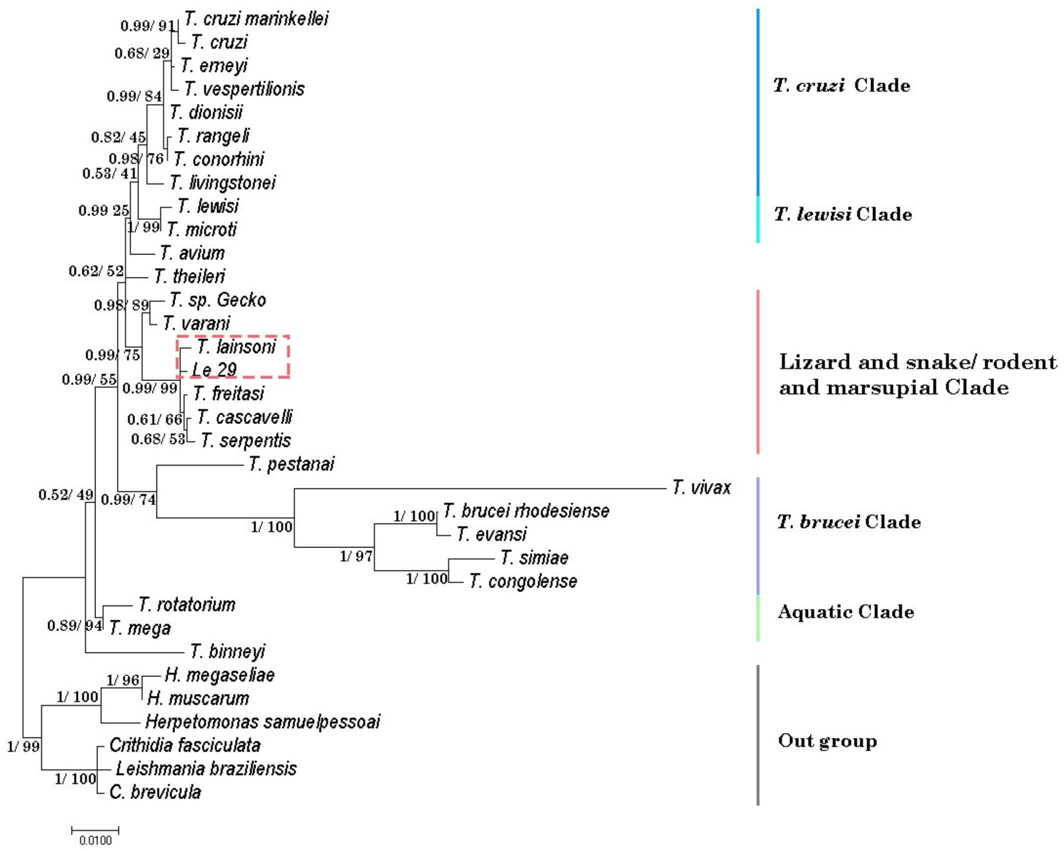

Figure S2. Phylogenetic reconstruction performed by the maximum likelihood method from 1431 bp of the 18S rDNA gene in 34 species of trypanosomatids. Species of the genera *Herpetomonas*, *Crithidia*, and *Leishmania* were included as out groups. The two values at the nodes represent: Bayesian analysis support/ML method bootstrap value. *Trypanosoma lainson*i and Le29 are found within the clade LSRM.
